# Isolated Large Glenoid Fracture in Acute Glenohumeral Dislocation in the Elderly: A Novel Indication for Reverse Shoulder Arthroplasty

**DOI:** 10.1155/2020/8826803

**Published:** 2020-08-12

**Authors:** Tyler Smith, Joseph D'Alonzo, Alfonso Arevalo, Jack Kazanjian

**Affiliations:** Department of Orthopedic Surgery, Philadelphia College of Osteopathic Medicine, 4190 City Ave, Philadelphia, PA, USA

## Abstract

**Case:**

Two elderly males presented with traumatic shoulder dislocation and bony Bankart fracture consisting of greater than 25% of the glenoid width. Due to several concomitant factors such as polytrauma, activity level, rotator cuff pathology, optimization of comorbidities, risk of complications, and potential for revision surgery, the patients were treated with reverse shoulder arthroplasty (RSA).

**Conclusion:**

RSA may be a satisfactory treatment option for isolated, large glenoid fractures associated with anterior glenohumeral instability in the elderly. These patients are susceptible to rapid deconditioning with prolonged immobilization and may not be medically suited to undergo the prolonged recovery period associated with open reduction internal fixation or potentially undergo revision operations.

## 1. Introduction

Bony Bankart fractures are a well-known complication of traumatic, anterior glenohumeral dislocation with an incidence ranging from 5.4 to 44% [1–5]. The majority are small and can be treated nonoperatively or with arthroscopic fixation and capsular repair [6–11]. Large Bankart fractures present a challenge to both patients and physicians as they are associated with a high risk of recurrent instability [12–15]. The gold standard treatment for these large fractures has traditionally been open reduction and internal fixation (ORIF); however, implant complications, recurrent instability, and revision surgery are all concerns [8, 10, 16–18]. Treating these injuries in the elderly patient is especially complicated given the high incidence of osteoporosis, rotator cuff atrophy, and rotator cuff injuries at the time of dislocation [7, 19–22]. In addition, elderly patients are especially prone to rapid deconditioning with prolonged immobilization and may not be able to adhere to strict weight-bearing restrictions after ORIF. We present two cases of acute, traumatic shoulder dislocation associated with large anteroinferior glenoid fractures treated with reverse shoulder arthroplasty (RSA). All patients provided informed consent for inclusion in the manuscript.

## 2. Case 1

**Figure 1:**
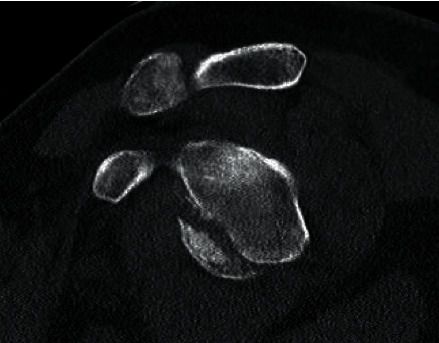
Sagittal CT image demonstrating large anteroinferior glenoid rim fracture involving approximately 25% of the glenoid width.

A 67-year-old male presented to the emergency department with multiple injuries including a left anteroinferior glenohumeral dislocation with associated glenoid fracture and Hill-Sachs lesion. The shoulder was found to be grossly unstable following closed reduction. The remainder of the patient's injuries were determined to be amenable to nonoperative treatment. CT confirmed an anterior glenoid fracture involving approximately 25% of the articular surface, with a concomitant large, Hill-Sachs lesion (Figure 1). Although nonoperative management was discussed with the patient, the size of the glenoid defect and signs of gross instability led to the decision of operative management. Isolated ORIF of the glenoid fracture was also considered, but given the patient's multiple injuries and weight-bearing deficiencies, it was felt that the postoperative restrictions following glenoid ORIF would be significantly functionally limiting. To optimize the patient's stability and to maximize his functional status immediately postoperatively, the patient was scheduled for RSA.

**Figure 2:**
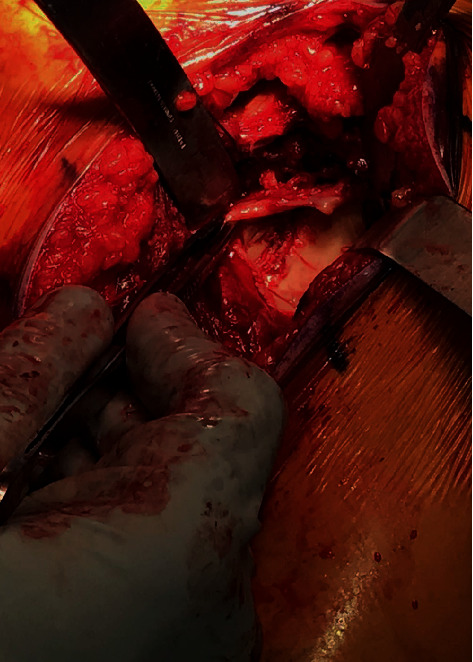
Clinical photo demonstrating exposure of bony Bankart fracture.

**Figure 3:**
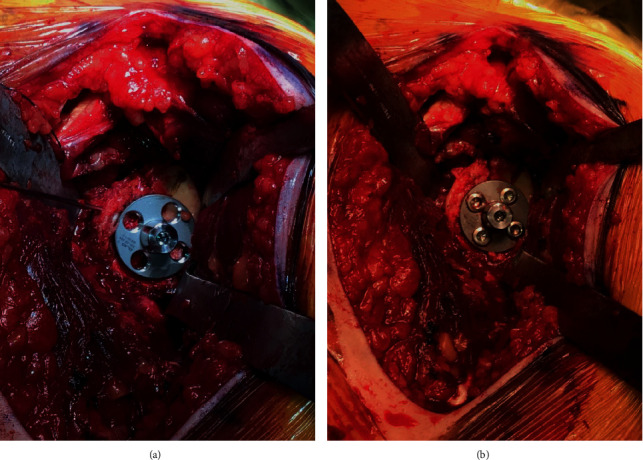
Clinical photos demonstrating provisional fixation with K-wires (a) and definitive fixation with compression of fracture fragments through the glenoid baseplate (b).

**Figure 4:**
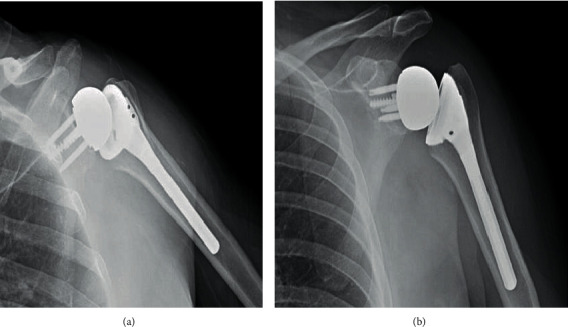
(a, b) Postoperative radiographs of Case 1 demonstrating reverse shoulder arthroplasty following fixation of large glenoid rim fracture via the baseplate.

Ten days after the initial injury, the patient was brought to the operating room. During exposure with a standard deltopectoral approach, a significant Hill-Sachs lesion and small rotator cuff tear were observed. Humeral head osteotomy was performed, and the anterior glenoid fracture was visualized and inspected (Figure 2). After reduction and provisional fixation of the glenoid fracture with K-wires, the glenoid was then prepared by drilling and tapping the central hole to accept the standard baseplate (Figure 3(a)). Special attention was directed toward the anterior screw to provide additional compression and fixation to the glenoid fracture fragment. The fragment was found to be stable and anatomically aligned following placement of the peripheral screws (Figure 3(b)). A neutral glenosphere was then attached, and the humerus was prepared with an acetabular reamer and sequential broaching. Final components were placed, and stability was confirmed. After three weeks in a sling, he began passive motion exercises. He was allowed to progress through full active range of motion by six weeks postoperatively. Radiographs obtained at that time demonstrated RSA in anatomic position with healing of the glenoid fracture through baseplate compression (Figures 4(a) and 4(b)). At one year, he is pain free, fully independent, and reports no disability or dysfunction in his daily activities.

## 3. Case 2

**Figure 5:**
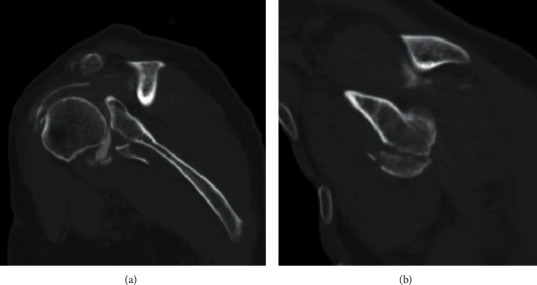
Coronal (a) and sagittal (b) CT images demonstrating comminuted bony Bankart fracture involving approximately 35% of the glenoid width.

A 66-year-old male presented to the emergency department after a fall resulting in a left glenohumeral dislocation and traumatic glenoid fracture. After successful closed reduction, examination revealed gross instability. Postreduction CT demonstrated a large, comminuted anteroinferior glenoid fracture involving approximately 35% of the glenoid diameter (Figures 5(a) and 5(b)). Nonoperative treatment was decided against due to the degree of instability and risk of recurrent dislocation. Isolated ORIF was discussed but decided against due to prolonged weight-bearing restrictions, immobilization, fracture comminution, risk of revision surgery, and the risk of posttraumatic arthritis and limited function.

Three days following his initial presentation, he returned to the operating room for RSA via a deltopectoral approach as discussed previously. Exposure of the glenoid revealed a comminuted fracture involving approximately 35% of the glenoid diameter. The fracture was reduced provisionally with K-wires prior to the placement of the glenoid baseplate. Similar to the patient in Case 1, the anterior screw of the baseplate was secured into the anteroinferior fracture fragment to allow for internal fixation within the compressed baseplate. Stability was confirmed, and final components were placed. He followed the same protocol as the patient in Case 1 and was allowed full active range of motion by six weeks postoperatively. At one-year follow-up, he has no pain and has returned to independent participation in his activities of daily living with minimal functional deficit.

## 4. Results

Both patients are now over one year from the initial injury and have returned to all independent activities. Active range of motion for Case 1 is forward elevation to 141 degrees, external rotation 0 to 35 degrees, and internal rotation to L2. Active range of motion for Case 2 is forward elevation to 145 degrees, external rotation from 0 to 41 degrees, and internal rotation to T10. The American Shoulder and Elbow Score (ASES) is 82 and 94 for Cases 1 and 2, respectively. The visual analogue scores (VAS) are 1 and 0, respectively. The Short Form 12 (SF-12) scores are 51.38 and 54.84, respectively. There were no complications for either patients since the date of surgery and the most recent follow-up visit at one year.

## 5. Discussion

Recurrent instability following glenoid fracture is directly related to the degree of bony deficit [12, 14, 23]. Itoi et al. demonstrated in a biomechanical study that fractures involving greater than 21% of the glenoid width are at high risk for recurrent instability even after repair [13]. The traditional treatment approach to acute large glenoid fractures has been ORIF. However, several studies have reported on the associated complications with ORIF [8, 16, 17, 24, 10]. Scheibel et al. reported on 10 patients with large, traumatic glenoid fractures involving more than 25% of the articular surface and found an early complication rate of 40% secondary to metal loosening and screw impingement. All complications in this study required revision ORIF [18]. Schandelmaier et al. evaluated outcomes of 22 patients with intra-articular glenoid fractures and found that 22.7% of patients developed radiographic evidence of posttraumatic osteoarthritis at mean follow-up of 5 to 10 years [24]. Additional concerns with isolated ORIF are risk of malunion, nonunion, revision surgery, prolonged immobilization, and postoperative weight-bearing restrictions. These considerations are especially important in the elderly patient due to the incidence of osteoporosis, poor bone stock, rotator cuff atrophy, and concomitant rotator cuff tears that occur at the time of dislocation [21, 22, 25–30]. While the two cases we discussed do not involve a significant rotator cuff injury, the incidence rotator cuff tears during instability are reported to be as high as 86% and may further confound the recovery after glenoid ORIF [7, 19–21, 28]. In addition, elderly patients with comorbid medical conditions may be susceptible to rapid deconditioning with prolonged immobilization and may not be medically suited for multiple operations.

The utilization of RSA for complex proximal humerus fractures in the elderly has increased dramatically over the past two decades due to predictable results in pain control and function; however, the literature regarding the treatment of large glenoid fractures in the elderly is limited [31–33]. Garofalo et al. performed a case series of 26 patients with large glenoid fractures associated with complex proximal humerus fractures treated with RSA and glenoid rim bone grafting. They reported good to excellent outcomes in 24/26 patients at an average of 36 months follow-up and no major complications or revision surgery [34]. Maassen et al. reported the use of RSA with concomitant glenoid screw fixation for a patient with a 4-part proximal humerus fracture and associated large glenoid fracture. They reported excellent pain control and good functional outcome at 1-year follow-up with full return to recreational activities and no complications or signs of instability [35].

Both cases in our series resulted in successful outcomes with the patents returning to full activity by 12 weeks and satisfactory clinical and functional scores at 1-year follow-up. Due to our successful experience with these 2 patients, we may consider accelerating the rehab protocol to allow unrestricted activity even sooner in future cases. The biomechanics of RSA allow for reliable restoration of activities in low-demand patients while decreasing and even avoiding many of the risks of glenoid ORIF. Fixation of the glenoid fragment through the baseplate provides 2 theoretical advantages: (1) distributing the force across a larger surface area and therefore reducing the strain on the screw and (2) providing a gentle compressive load to the glenoid fracture. Combining modified ORIF with RSA also allows for treatment of associated pathology, rotator cuff injuries, rotator cuff atrophy, maintenance of shoulder stability, a simplistic postoperative rehabilitative program, and early pain relief and functional return. Although there is limited data on this topic, RSA may be a reasonable option for the treatment of large glenoid fracture with recurrent instability in the elderly.
